# Conceptualization of a cognitively enriched walking program for older adults: a co-design study with experts and end users

**DOI:** 10.1186/s12877-022-02823-z

**Published:** 2022-03-01

**Authors:** Pieter-Jan Marent, Arwen Vangilbergen, Sebastien Chastin, Greet Cardon, Jannique G. Z. van Uffelen, Melanie Beeckman

**Affiliations:** 1 Department of Movement Sciences, KU Leuven, Leuven, Belgium; 2 Department of Movement and Sports Sciences, Ghent University Research Centre for Aging Young, Ghent University, Ghent, Belgium; 3 School of Health and Life Science, Glasgow Caledonian University, Glasgow, UK

**Keywords:** Intervention, Prevention, Physical activity, Cognition

## Abstract

**Background:**

Research in controlled laboratory settings shows that physical activity programs enriched with cognitive challenges enhance the benefits of physical activity on cognition in older adults. This translational study aimed to conceptualise a real-life, cognitively enriched walking program for older adults (65+) by investigating (a) which cognitive tasks are most suited for cognitive enrichment of a walking program, and (b) how to embed these tasks in a walking program to become feasible, enjoyable and effective.

**Methods:**

A co-design process was followed with input of 34 academic experts and 535 end users. First, an online, three-rounds Delphi process was used to obtain consensus amongst academic experts on the key characteristics that a real-life cognitively enriched walking program should have. Next, end users provided feedback and suggestions on what the experts concluded, and gave more insight into their preferences and concerns by means of an online/telephone survey.

**Results:**

Combined input from experts and end users resulted in a list of recommendations to guide the further development of the cognitively enriched walking program. First, it is important to provide a range of cognitive tasks to choose from. Each of these tasks should (a) provide variation and differentiation, (b) be implemented with increasing levels of difficulty, and (c) be integrated in the walk. Second, divide the walk into three parts: 5–10 min brisk walking, cognitive tasks for most of the walk, and 5–10 min free walking. Finally, the program should strive for a minimal session frequency of twice a week, include competition occasionally and carefully, ensure safety and keep the walks fun.

**Conclusions:**

The co-design process resulted in recommendations to guide the next steps in the program development process. Additional studies will be performed to improve the enjoyability and feasibility, and to assess the effectiveness of the cognitively enriched walking program to improve cognitive functioning and physical activity in older adults (65+).

**Supplementary Information:**

The online version contains supplementary material available at 10.1186/s12877-022-02823-z.

## Background

Increased longevity is one of the greatest success stories in the field of public health. Nevertheless, additional life years are not always lived in optimal health. Aging is not only associated with a strong increase in the prevalence of chronic and degenerative diseases and functional impairments, but also with a decline in several cognitive abilities, such as processing speed, memory and executive function [1, 2]. Retaining optimal cognitive health is crucial, as it strongly associates with physical functioning and the ability to live independently [3]. Cognitively healthy older adults rely less on their family for day-to-day assistance, need fewer medical services and incur fewer medical costs, resulting in a more sustainable health care system [4]. Consequently, retaining optimal cognitive health not only drastically improves older adults’ quality of life, but also benefits their families and society in general [4].

Physical activity (PA), defined as any bodily movement produced by skeletal muscles that results in energy expenditure [5], as well as other modifiable lifestyle factors such as cognitive engagement and diet, can have a beneficial impact on cognitive health during aging [6]. A recent meta-analysis of 33 randomized controlled trials in adults aged 50 years and above showed significant gains in cognitive function following a physical exercise intervention versus no intervention (SMD = 0.29; 95% CI 0.17 to 0.41) [7]. This improvement is, in part, the result of PA facilitating neural plasticity by upregulating key growth factors (e.g. Brain-Derived Neurotrophic Factor), increasing angiogenesis and neurogenesis, and preserving and increasing grey and white matter volumes [8].

Lately, evidence has emerged for even larger cognitive gains after PA-interventions that are combined with cognitive activities (CA) [9–11]. For instance, the meta-analysis of Zhu et al. (2016) found a significant difference in overall cognition between interventions combining physical exercise and cognitive activity and those with physical exercise only (SMD = 0.22; 95% CI 0.06 to 0.38). However, the combined training was not more effective than cognitive training alone [9]. It is argued that combining physical and cognitive activity (PA + CA) has a synergistic impact on brain plasticity: whereas PA facilitates plasticity, additional CA promotes the survival of the newly-formed neurons and its functional integration into the existing neural networks [10, 12]. Most studies examined the effect of combined PA + CA under controlled laboratory conditions using dual-tasking paradigms or exergames [9–11]. However, an important shortcoming of current research is that PA + CA programs have yet to be tested in real-life settings. There is a need for translational research developing effective and feasible, real-life PA + CA programs which are sustainable, scalable and easily accessible to the majority of the older population.

The current study aims to conceptualise a real-life, cognitively enriched walking program for older adults (i.e. aged 65 years and above). Walking is the most common type of PA amongst older adults [13] and a well-established mode of PA intervention in this population [14]. Moreover, walking allows for social interaction and is convenient, affordable, safe, independent of exercise facilities and easy to integrate in daily life activities; which are all key determinants of motivation and long-term adherence to PA programs for older adults [15–18].

Despite the promising effects of PA + CA interventions in controlled laboratory settings [11], explicit guidelines on the essential characteristics of a real-life, cognitively enriched walking program are lacking. Therefore, this study introduced an innovative co-design methodology with academic experts and end users (i.e. potential program participants and coaches) to conceptualise the first, real-life, cognitively enriched walking program for older adults. Two research questions were addressed: (a) which cognitive tasks are most suited for cognitive enrichment of a walking program for older adults; and (b) how to embed these tasks in a walking program to become feasible, enjoyable and effective.

## Methods

For the co-design of the program, two groups of interest (i.e. academic experts and end users) provided input. First, an expert panel sought consensus on key characteristics of a real-life, cognitively enriched walking program (e.g. targeted cognitive functions, frequency of the program). Next, end users provided feedback and suggestions on what the experts concluded, and expressed their personal preferences and concerns. The study was conducted in accordance with the Declaration of Helsinki and with the positive approval of the Ethical Committee of Ghent University Hospital (EC/2019/1045) and the Ethical Committee Research KU/UZ Leuven (S63305).

### Input from academic experts – Delphi study

Experts’ input was sought by means of a predefined three-rounds Delphi process. This iterative multistage methodology facilitates the gathering of individual opinions in a systematic manner and the synthetization of these opinions into a group consensus [19, 20].

#### Participants

Purposive sampling was applied for the recruitment of a diverse panel with expertise in neuroscience, physical activity, (neuro)psychology and aging. Potential experts were selected from the researchers’ existing networks and from peer-reviewed papers on cognitively enriched PA programs. In addition, snowball sampling was used by asking the respondents in the first and second round to suggest other relevant experts. After verifying their expertise, additional experts were invited to participate in Round 2. For the final round, no new experts were invited. For each round, an online survey link was emailed to the experts with a reminder after 1 week. To be included, experts were asked to give their informed consent, and some personal characteristics (i.e. gender, age, academic rank, areas of expertise, and years of relevant expertise). Anonymity of the respondents was maintained throughout all three rounds.

#### Delphi round 1

The aim of the first round (January 2020) was to acquire an extensive overview of (a) the specific cognitive functions to target in order to optimally boost neuroplasticity in older adults, (b) the most favourable characteristics of a cognitively enriched walking program (i.e. cognitive load, frequency of the walks) and (c) concrete examples of cognitive tasks. Therefore, six open-ended questions covering these three topics were presented to a limited panel of key experts (see Additional file 1). Fifty experts were invited, of which 23 consented to participate (i.e. response rate: 46%). Twenty-two completed the survey and were included in the analyses. Content analysis was performed to structure and quantify the answers to the open-ended questions. The results of Round 1 formed the basis for the questions and answer options in Round 2.

#### *Delphi round* 2

The aim of this round (February 2020) was to refine the information provided by the experts in the first round. A larger group of experts were asked to rate the importance of the responses in Round 1 by selecting their top three choices from a list. They were provided with frequency distributions of the first round’s results and were asked to respond to six close-ended questions on the type of cognitive functions to target and the characteristics of the program (i.e. cognitive load and frequency) (similar to the questions asked in Round 1, see Additional file 2). In addition, the option was given to provide concrete examples of cognitive tasks, explain answers, or leave comments. This allowed new experts to provide additional input when disagreeing with the given answer options. A total of 112 experts were invited of which 34 consented to participate and provided complete data (i.e. response rate: 30%; 52.9% also participated in Round 1). Descriptive analyses were conducted to identify the most chosen answer options. The results served as the framework for the concluding statements of Round 3.

#### Delphi round 3

The aim of this final round (March 2020) was (a) to gain consensus on the type of cognitive functions to target and the characteristics of the program (i.e. cognitive load and frequency); and (b) to receive feedback on the specific cognitive tasks proposed in the first two rounds. These tasks were categorized in three matrices, representing different types of tasks (see Additional file 3). Experts received the results from Round 2 and were asked to indicate their level of agreement on six concluding statements on a 5-point Likert scale (i.e. strongly disagree, disagree, neither agree nor disagree, agree, strongly agree) or choose the option ‘I do not wish to answer’. Experts’ opinions on the potential effectiveness and feasibility of the cognitive tasks were assessed with a yes/no question (with also the option ‘I do not wish to answer’). If the response was ‘no’, the expert was asked for an explanation. Ninety-six experts were invited of which 28 consented to participate (i.e. response rate: 29%). One expert dropped-out after giving consent. Twenty-seven experts were included in the analyses (74.1% participated in Round 1 and 2; 25.9% only in Round 2). Descriptive statistics were conducted to identify consensus, which was achieved when agreement was 70% or higher (i.e. experts chose ‘agree’ or ‘strongly agree’) [21].

### Input from end users – survey

The next step in the co-design process was to gather end users’ opinion about (a) the specific cognitive tasks suggested by the experts and the overall cognitively enriched walking program, and (b) the preferred program characteristics. Therefore, two groups of end users were identified: “participants”, defined as adults aged 60 years and above who are able to walk independently; and “coaches”, defined as adults of any age who organize, lead or guide an organized group walking program for older adults.

#### Grouping of cognitive tasks

First, overlapping and compatible cognitive tasks, derived from the Delphi study, were grouped. For instance, “Verbal fluency task”, “Name animals starting with the last letter from the animal mentioned by the previous person”, and “Name countries with a particular letter” were grouped into *“Words starting with a particular letter”.* Next, each group of cognitive tasks was further elaborated to provide variation and differentiation in terms of complexity, individual- versus group-performance, and competition versus teamwork. For instance, the grouped task *“Words starting with a particular letter”* contained different variations such as describing as many words as possible in 1 minute, naming one word as fast as possible, taking turns for naming a word while avoiding to name a word that has already been mentioned, creating a word chain by naming words that start with the last letter from the previous word, and choosing categories to which each word should belong. Finally, each group of cognitive tasks was provided with a title, a short written instruction (to be found in Additional file 4) and a one-minute instruction video to ensure the nature of the task was clear to the end users.

#### Procedure

A survey was conducted to collect end users’ opinions. Therefore, a convenience sample was recruited through an online flyer distributed via group walking programs for older adults, organizations for older adults, and social media in Flanders (Belgium). The flyer contained two options: a link to complete the survey online, and a telephone number for those who preferred to answer the questions over the phone. For the phone interviews, a research assistant opened the online survey, read aloud the questions, and filled in the response. At the start of the survey, an estimated administration time of 30 to 60 min was mentioned, and an informed consent could be signed by means of checkboxes. All data were collected between April and May 2020.

#### Survey

First, the survey captured several sociodemographic variables; age, gender, country of birth, educational level, marital state, employment, financial state and PA level (see Table 1). Additionally, end users’ affinity with organized group walking programs for older adults was assessed; end users who indicated to organize, guide or lead such a program were seen as ‘coaches’, all others were seen as ‘participants’. Second, instructions were provided for each (group of) cognitive tasks; a short written-instruction and a one-minute video in the online survey; and a short oral-instruction and the possibility to ask questions in the phone interview. End users rated the enjoyability (i.e. fun to perform) and feasibility (i.e. practically possible) of the specific (groups of) cognitive tasks and the overall cognitively enriched walking program on 3-point scales (0 ‘not enjoyable/feasible’; 1 ‘somewhat enjoyable/feasible); 2 ‘enjoyable/feasible’). They could also provide explanations for their ratings and suggestions for improvement in open-ended questions. To limit the length of the survey and increase the response rate, the cognitive tasks were divided into three sets so that end users could choose to rate one, two, or all sets. Next, end users’ preferred program characteristics were assessed (i.e. cognitive load, duration, frequency and the inclusion of competition) (see Table 2). An open-ended question was provided to explain their ratings for competition.

**Table 1:** Characteristics of end users who participated in the survey

	Participants		Coaches	
	*N* (%)		*N* (%)	
	(*n = 468*)		(*n = 67*)	
Administration				
Online	460	(98.3)	67	(100)
Telephone	8	(1.7)	0	(0.0)
Gender *(What is your gender?)*				
Male	205	(43.9)	39	(58.2)
Female	262	(56.1)	28	(41.8)
Country of birth *(In which country were you born?)*				
Belgium	440	(94.2)	63	(94.0)
The Netherlands	11	(2.4)	1	(1.5)
Other (Congo, Germany, France, USA, Spain, Italy, Rwanda)	16	(3.4)	3	(4.5)
Educational level *(What is your highest diploma or certificate?)*				
No degree	2	(0.4)	0	(0.0)
Primary education or lower	13	(2.8)	1	(1.5)
Secondary education	118	(25.2)	24	(35.8)
Higher education, non-university	224	(47.9)	34	(50.7)
University education	111	(23.7)	8	(11.9)
Financial state *(How would you describe your current financial situation?)*				
Difficult	3	(0.6)	0	(0.0)
Rather difficult	10	(2.1)	1	(1.5)
Sometimes easy, sometimes difficult	77	(16.5)	10	(14.9)
Rather easy	174	(37.2)	28	(41.8)
Easy	192	(41.0)	26	(38.8)
I do not wish to answer	12	(2.6)	2	(3.0)
Employment *(Which is correct regarding your employment state?)*				
At work	60	(12.8)	17	(25.4)
Retired	358	(76.5)	47	(70.1)
Partially retired	21	(4.5)	1	(1.5)
Incapacitated for work	8	(1.7)	1	(1.5)
Homemaker/housewife	13	(2.8)	0	(0.0)
Other	8	(1.7)	1	(1.5)
Marital state *(What is your marital state?)*				
Single	30	(6.4)	6	(9.0)
In a relation, not living together	12	(2.6)	5	(7.5)
In a relation, living together	26	(5.6)	2	(3.0)
Married	330	(70.7)	47	(70.1)
Divorced	36	(7.7)	4	(6.0)
Widower/widow	32	(6.9)	3	(4.5)
Other	1	(0.2)	0	(0.0)
Physical activity level *(Are you physically active at least five times a week for 30 min or more each day?)*				
Almost never	14	(3.0)	2	(3.0)
Usually not	45	(9.6)	5	(7.5)
Sometimes yes, sometimes not	93	(19.9)	18	(26.9)
Usually yes	148	(31.6)	20	(29.9)
Almost always	168	(35.9)	22	(32.8)
Involvement in an organized group walking program *(Are you involved in an organized group walking program for older adults?)*				
No, I rarely walk	45	(9.6)	/	/
No, but I walk regularly on my own	118	(25.2)	/	/
No, but I walk regularly with friends and/or family	177	(37.8)	/	/
Yes, I participate in an organized group walking program for older adults	128	(27.4)	/	/
Yes, I organize, lead or guide an organized group walking program for older adults	/	/	67	(100)
Age, mean (*SD*), years	68.4	(5.8)	65.4	(10.5)

Notes: *N*(%) number of end users (percentage), *SD* standard deviation

**Table 2:** End users’ preferred program characteristics

	Participants		Coaches	
	*N* (%)		*N* (%)	
	(*n = 468*)		(*n = 67*)	
Cognitive load *(How long would you be willing to perform cognitive tasks during a 30-min walk?)*				
No cognitive tasks at all	92	(20.5)	17	(26.6)
0–5 min	28	(6.2)	6	(9.4)
5–10 min	60	(13.4)	27	(26.6)
10–15 min	106	(23.6)	12	(18.8)
15–20 min	83	(18.5)	6	(9.4)
20–25 min	22	(4.9)	6	(9.4)
25–30 min	58	(12.9)	0	(0.0)
Duration *(How long do you think such a cognitively enriched walk should take (not counting any rest breaks)?*				
Less than half an hour	41	(9.1)	6	(9.4)
Half an hour	60	(30.4)	10	(15.6)
One hour	192	(42.8)	27	(42.2)
One hour and a half	67	(15.1)	8	(12.5)
Two hours	63	(14.0)	10	(15.6)
More than two hours	25	(5.6)	3	(4.7)
Frequency *(How often would you be willing to participate in such a cognitively enriched walking program?)*				
Never	114	(25.4)	18	(28.1)
Less than once per week	86	(19.2)	15	(23.4)
Once per week	174	(38.8)	22	(34.4)
Two times per week	44	(9.8)	6	(9.4)
Three times per week	17	(3.8)	2	(3.1)
Four times per week	6	(1.3)	0	(0.0)
Five times per week	7	(1.5)	1	(1.6)
Six times per week	0	(0.0)	0	(0.0)
Seven times per week	1	(0.2)	0	(0.0)
Competition *(What do you think of a competitive aspect in the cognitive tasks?)*				
Enjoyable	173	(38.5)	22	(34.3)
Neither ‘enjoyable’ nor ‘not enjoyable’	138	(30.7)	22	(34.3)
Not enjoyable	138	(30.7)	20	(31.3)

Note*: N*(%) number of end users (percentage)

#### Analyses

SPSS 26 was used for all quantitative analyses. Sample characteristics were described with descriptive statistics. Enjoyability and feasibility ratings for the cognitive tasks and the overall program were averaged to calculate suitability scores, which give an indication on how convenient the tasks and the program are for implementation and sustainability. After all, while participants should find it enjoyable to do, it should also be feasible to perform and vice versa. This score ranges from zero (not enjoyable and not feasible) to two (enjoyable and feasible). Descriptive statistics were used to rank the cognitive tasks from most to least suitable and to describe the preferred program characteristics.

Inductive content analyses were performed on the qualitative data comprising explanations for the ratings and suggestions for improvement. First, three reviewers independently coded the answers of three different open-ended questions. Then, these codes were discussed and a code book was composed. The code book contained a coding tree, and for every code a definition and an example. Third, the answers of two other open-ended questions were coded by two of the three reviewers. The reviewers discussed and finalized the code book. Finally, the code book was applied to code all open answers in Microsoft Excel. Importance was gauged using frequency of occurrence of each code.

## Results

### Input from academic experts – Delphi study

The characteristics of the respondents from each round are presented in Table 3. There was consensus for all six concluding statements on the type of cognitive functions to target (i.e. executive function and memory) and the characteristics of the program (Table 4). Additionally, it was indicated that “episodic memory” should be added to the statement concerning the specification of “memory and learning”.

**Table 3:** Characteristics of experts in the Delphi study

	Round 1		Round 2		Round 3	
	*N* (%)		*N* (%)		*N* (%)	
	(*n =* 22)		(*n =* 34)		(*n =* 27)	
Gender, Male	13	(59.1)	16	(47.1)	14	(51.9)
Academic rank						
Professor	15	(68.2)	26	(76.5)	19	(70.4)
Other academic position ^a^	7	(31.8)	8	(23.5)	8	(29.6)
Expertise						
Psychology	3	(13.6)	10	(29.4)	5	(18.5)
Physical activity & Movement Sciences	9	(40.9)	13	(38.2)	17	(63.0)
Cognition & Neuroscience	13	(59.1)	18	(52.9)	14	(51.9)
Aging & Older adults	9	(40.9)	10	(29.4)	10	(37.0)
Other	1	(4.5)	3	(8.8)	2	(7.4)
Age, mean (*SD*), years	48.7	(12.0)	48.8	(11.4)	47.6	(11.9)
Years of relevant expertise, mean (*SD*)	21.2	(12.7)	21.4	(10.9)	20.4	(11.5)

Notes*: N*(%) number of respondents (percentage), *SD* standard deviation

^a^ PhD, Senior researcher, Research associate/fellow/scientist, Postdoc, Privatdozent

**Table 4:** Agreement on concluding statements ^a^ in round 3 of the Delphi study

	% agree ^b^
Targeted cognitive functions	
The PA + CA program should primarily target executive functioning and higher-order thinking, as well as memory and learning.	77.7
With regard to executive functioning and higher-order thinking, the program should mostly focus on working memory, cognitive inhibition/inhibitory control, cognitive flexibility and planning.	88.9
With regard to memory and learning, the program should mostly focus on the visuospatial and verbal/phonological loop of the working memory.	74.1
The most important reason in selecting the targeted cognitive functions for the PA + CA-program is their real-life relevance.	81.4
Cognitive load	
During the 30-min walk, minimum 15–20 min should be allocated to cognitive activities in order to improve cognitive function.	96.2
Frequency	
During the week, the PA + CA program should be organized at least 2–3 times in order to improve cognitive function.	92.6

Note*: PA + CA* Physical Activity and Cognitive Activity

^a^ Round 3 wording presented

^b^ Percentage of respondents (*n* = 27) answered ‘agree’ or ‘strongly agree’

Throughout the first two rounds, experts provided 134 examples of cognitive tasks, which were then merged and presented in Round 3 in three matrices containing 52 different types of tasks. Only four proposed tasks were found unsuitable due to feasibility reasons, namely the “Flanker Task”, “Wisconsin Card Sorting Task”, “Attention Network Task” and the “Auditory Continuous Performance Task” (see Additional file 5). All other cognitive tasks were considered effective and feasible according to the experts in Round 3 (see Additional files 6, 7, 8).

### Input from end users – survey

#### Sample characteristics

Data from 535 end users were included for analyses (67 coaches and 468 participants; 527 online survey and 8 phone interview). The data from 316 others were excluded because they were younger than 60 years (*n* = 16), were not able to walk independently (n = 1) or dropped out before finishing the assessment of at least one set of cognitive tasks (*n* = 299). See Additional file 9 for a flowchart and Table 1 for the sociodemographic characteristics of the included participants and coaches.

#### Rating of the specific cognitive tasks and the overall cognitively enriched walking program

The ratings for the 32 (groups of) cognitive tasks are presented in Table 5. The mean suitability score for the program was 1.42 (±0.68) on a scale from zero to two. The percentage of end users that expected the program to be enjoyable and feasible was 57.1 and 60% respectively.

**Table 5:** Rating of the (groups of) cognitive tasks ^a^

Cognitive tasks	Participants					Coaches				
	N	Enjoyable (%)	Feasible (%)	Suitability Mean (SD)		N	Enjoyable (%)	Feasible (%)	Suitability Mean (SD)	
1. Facts and titbits	231	79.2	82.3	1.75	0.51	33	72.7	69.7	1.59	0.69
2. Quest with environmental clues	205	82.4	82.9	1.76	0.54	28	64.3	67.9	1.52	0.73
3. Awareness	183	68.9	77.0	1.63	0.62	24	83.3	79.2	1.81	0.36
4. Spotted	183	74.9	73.2	1.66	0.58	24	75.0	62.5	1.58	0.64
5. Opinions	205	65.4	78.0	1.64	0.56	28	82.1	75.0	1.61	0.75
6. Notice and remember symbols	231	68.8	71.4	1.61	0.61	33	63.6	60.6	1.47	0.73
7. Quiz	205	68.3	71.2	1.60	0.63	28	71.4	67.9	1.52	0.78
8. Plan the route	231	74.0	68.0	1,60	0.63	33	60.6	48.5	1.42	0.63
9. Quest	183	74.9	68.9	1.60	0.65	24	62.5	54.2	1.38	0.81
10. Hidden word	231	70.1	72.3	1.60	0.63	33	54.5	48.5	1.26	0.80
11. Words starting with a particular letter	231	64.9	68.8	1.55	0.62	33	51.5	57.6	1.33	0.76
12. Problem solving	231	66.2	63.2	1.55	0.62	33	48.5	48.5	1.27	0.76
13. Word associations	205	64.4	73.2	1.55	0.66	28	46.4	46.4	1.16	0.83
14. Remember the route	205	63.9	61.0	1.50	0.61	28	60.7	53.6	1.43	0.68
15. I spy	205	62.9	68.3	1.50	0.69	28	46.4	50.0	1.25	0.79
16. A new language	231	63.2	60.6	1.48	0.68	33	39.4	39.4	1.12	0.78
17. Buzz it	183	53.0	56.8	1.37	0.71	24	62.5	54.2	1,44	0,73
18. Story telling	205	55.1	58.5	1.37	0.73	28	46.4	46.4	1.13	0.86
19. Geocaching	231	55.4	51.1	1.39	0.75	33	54.5	39.4	1.11	0.85
20. Serial subtraction task	231	51.9	61.0	1.36	0.73	33	36.4	45.5	1.06	0.80
21. Memory techniques	183	48.1	50.3	1.32	0.70	24	50.0	50.0	1.29	0.79
22. Music	183	55.2	53.6	1.32	0.75	24	50.0	50.0	1.19	0.75
23. Obstacle walk	183	52.5	53.0	1.30	0.77	24	41.7	37.5	1.06	0.81
24. Order of daily activities	205	48.3	46.8	1.24	0.75	28	42.9	39.3	1.04	0.88
25. Mental arithmetic	205	45.4	51.2	1.23	0.77	28	28.6	28.6	0.79	0.82
26. The alphabet	183	41.5	42.1	1.15	0.73	24	37.5	33.3	1.08	0.76
27. Choreography	183	43.2	46.4	1.14	0.78	24	41.7	45.8	1.10	0.86
28. Immediate recall	183	37.7	45.9	1.13	0.74	24	33.3	45.8	1.06	0.78
29. N-back	231	39.0	41.6	1.14	0.74	33	33.3	39.4	0.95	0.85
30. List learning	205	36.1	40.5	1.13	0.72	28	25.0	28.6	0.91	0.76
31. Stimulus-response	205	39.0	39.5	1.10	0.73	28	28.6	32.1	0.88	0.79
32. Ball-games	231	40.7	35.9	1.06	0.78	33	48.5	21.2	0.98	0.71

Notes*: N*(%) number of participants (percentage), *SD* standard deviation

^a^ Tasks are ranked from most to least suitable based on the scores of all end users. Descriptions of the tasks can be found in Additional File 4

#### Explanations for the ratings and suggestions for improvements

Reasons for rating the tasks and/or the program as not enjoyable and/or not feasible were (a) personal preferences (e.g. “I just do not like this task”), (b) disruption of walking goals such as rest and relaxation (e.g. clear the mind and enjoy the surroundings), social interaction (e.g. spontaneous small talk) and physical training (e.g. brisk walking pace), (c) disruption of habits (e.g. walking alone), (d) threats related to group dynamics (e.g. risk of quarrels), (e) not seeing the benefits of performing cognitive tasks while walking, and (f) perceiving the tasks as “childish”, “artificial”, or “school-like”.

Reasons for rating the tasks and/or the program as enjoyable and feasible were (a) personal interest (e.g. “I just like this task”), (b) personal experience or skills (e.g. “I am good at this kind of task”), (c) group-specific opportunities (e.g. “This task might induce a positive group atmosphere because it stimulates to get to know and help each other”), (d) cognitive benefits (e.g. “This task seems to be a good memory training”), (e) opportunities to learn new things (e.g. “We will learn new words during this task”), and (f) making the walk more enjoyable, interactive, interesting or varied.

Finally, participants gave suggestions for improvement: (a) perform the tasks in small groups of about five people, (b) provide a positive group atmosphere to include and challenge everyone, (c) slowly increase complexity levels and adapt them to individual capabilities to ascertain an optimal level of challenge, (d) choose a suitable and safe location, (e) integrate the tasks into the walk so that it makes sense to perform them while walking, (f) consider performing the tasks during a break for the oldest participants or the ones with high fall-risk, (g) do not overload the coaches by minimizing the amount of preparations and materials, and (h) try to include (grand)children in the cognitively enriched walks.

#### Program characteristics

End users’ preferred program characteristics are described in Table 2. The majority of end users preferred a cognitive load of 10 to 15 min per 30 min of walking (*n* = 118, 23.0%), a walking duration of 1 h (*n* = 219, 42.7%) and a frequency of once per week (*n* = 196, 38.2%). More than 20% of end users reported to be not willing to participate in a cognitively enriched program at all (*n* = 132, 25.7%), or to perform any cognitive tasks while walking (*n* = 109, 21.2%).

There was no clear tendency for or against the inclusion of competition. Reasons for rating competition as ‘not enjoyable’ were (a) the mismatch between walking and competition (e.g. walking should be relaxing, pleasant and enjoyable), (b) the negative attitude towards competition in general, (c) the negative influence of competition on group dynamics (e.g. provoking strife, hostility, arrogance and know-it-all behaviour), (d) the negative feelings associated with losing, (e) the expectation that the same people will always win, (f) the feeling that there is already enough competition in the society, (g) the risk that people who lose often will drop out, and (h) the introduction of unnecessary stress to perform. Reasons for rating competition as ‘enjoyable’ were (a) the positive attitude towards competition in general, (b) the believe that competition can be a good motivator to do your best, and (c) the thought that competition can give the walk an extra dimension (e.g. excitement). Additionally, end users gave suggestions on how to include competition: (a) create a positive, light-hearted atmosphere, (b) include competition occasionally, but not too often, (c) make sure that the group is not too large and the participants are well-matched, (d) make good agreements with the participants, (e) consider the use of competition between teams instead of between individuals, and (f) encourage participants to strive for a certain standard instead of striving against each other.

## Discussion

This study aimed to conceptualise for a real-life, cognitively enriched walking program for older adults, using a co-design process with academic experts and end users.

### Cognitive tasks

The experts concluded that the cognitive tasks, used for cognitive enrichment of a walking program for older adults, should primarily target executive functioning and higher-order thinking as well as memory and learning. This is in line with the literature stating that these cognitive abilities are more susceptible for age-related decline as well as benefit the most from an intervention [7, 11, 22]. The cognitive tasks the experts agreed on targeted these cognitive functions and were also considered feasible for cognitive enrichment of a walking program.

When presented to the end users, some cognitive tasks were seemingly preferred over others. For instance, a quiz scored better than learning lists (see Table 5). Despite the aim to only select the most optimal cognitive tasks for the program, it appeared to be more beneficial to include all tasks. First, the experts agreed that all tasks were suitable and some of them explicitly mentioned that providing a wide range of tasks would yield better results than focusing on a few (i.e. better mimicking the real-world, increasing chances of far transfer effects, and preventing the program from becoming monotonous). Second, including a range of tasks will increase the chances of meeting everyone’s preferences as it became apparent in the survey that end users sometimes had divided opinions on certain tasks. And third, literature on self-determination theory shows that providing a pool of different tasks to choose from will introduce freedom of choice, which will support the basic psychological need for autonomy, and thus, increase autonomous motivation [23]. Therefore, all cognitive tasks will be included in the program.

Refinement and further elaboration of the cognitive tasks was needed after considering the comments and suggestions from the experts and end users, especially for cognitive tasks that received low suitability scores. More specifically, tasks were integrated better in the walk so that performing them while walking would feel more natural. For instance, for the cognitive task ‘obstacle walk’, it was recommended to use natural obstacles (e.g. a fallen tree) instead of artificial objects. In addition, different and increasing complexity levels were provided for every cognitive task to make the program sufficiently feasible and challenging for every individual, allowing for interindividual differences in terms of cognitive and physical functioning. For instance, index cards were developed for the cognitive task ‘hidden word’ containing five different words ranked from easy to difficult. If a participant successfully describes the first (easiest) word, they will be encouraged to describe the second (more difficult) word. This is consistent with the review of Wollesen (2014) stating that the cognitive challenge should be appropriate for each individual and should have increasing demands in order to gain better cognitive effects [24]. Adapting the program to participants’ individual cognitive and physical competences is also important to avoid frustration and drop out. Next, more variation was created within the tasks. For instance, in one variation of the task ‘noticing and remembering symbols’ photos were used that refer to an overarching theme (e.g. a local celebrity, a historical event). Thus, participants do not only have to search and remember the symbols, they also have to figure out the overarching theme. Finally, better framing of the cognitive tasks was added to increase participants understanding of the usefulness and potential benefits of each task.

### Program characteristics

Recommendations were formulated on the characteristics of the program. First, the cognitive load during the walk should be sufficient, but not too high. The experts recommended that minimum 15–20 min should be allocated to cognitive tasks during a 30 min’ walk. However, they mentioned that more research is needed to find strong, scientific grounds for the precise dose. Most end users stated to be willing to perform cognitive tasks during a walk, as long as there is still enough time to fulfil other walking goals (e.g. rest and relaxation, spontaneous small talk, brisk walking).

Second, the walk should take approximately 30–60 min depending on the physical capabilities of the participants. This is a common duration for walking programs in Flanders (Belgium) [25] and, according to the survey, this duration should be feasible for more than 90% of the end users. Moreover, exercise interventions of 45–60 min were associated with cognitive improvements in the meta-analysis of Northey et al. (2018), while shorter (< 30 min) and longer (> 60 min) ones were not [7]. In addition, they found that the exercise intervention should at least be of moderate intensity. This finding is in line with the concerns of some experts and end users in the survey, i.e. to keep an eye on walking pace as walking pace, and thus intensity, might decrease due to the simultaneous execution of cognitive tasks. In fact, a meta-analysis of Smith et al. (2015) found a reduction in gait speed under dual-task conditions in older adults [26]. A few minutes of brisk walking before engaging in the cognitive tasks can be used to increase participants’ heart rate. Thus, the walk can be divided into three parts: (a) a warm-up of (brisk) walking for 5–10 min; (b) cognitive tasks for a larger part of the walk (approximately 15–20 min per 30 min of walking); (c) spontaneous small talk or rest and relaxation for the last 5–10 min.

Third, the experts agreed that the program should be organized at least twice a week to potentially obtain positive effects on cognition, which echoes previously reported findings [24]. However, the meta-analysis of Gheysen et al. (2018) found no significant influence of session frequency on the cognitive effects of PA + CA interventions [10]. As the precise dose-response relationship remains unclear, some experts proposed to give the participants as many occasions to engage in PA + CA as possible. However, the minimal frequency of twice a week does not coincide with the preferred frequency of the end users since only a minority of them indicated to be willing to participate twice a week or more. Moreover, about a quarter of end users reported not to be willing to participate in a cognitively enriched walking program at all. It is therefore of utmost importance to (a) address the current concerns of the end users and take up their suggestions; and (b) improve the attractiveness of the program by implementing motivational strategies as described in the systematic review of reviews of Zubala et al. (2017) on the promotion of PA interventions for community dwelling older adults [14]. Furthermore, participants should receive the option to engage – alone or with friends/family – in cognitively enriched walks outside the organized walking sessions (e.g. by a home program).

Fourth, it is recommended to include occasional competition, but only if the participants are open for it. Previous research showed that competition can be a motivating factor for many older adults to engage in different types of games and to challenge themselves to do better [27]. Indeed, this study confirms that friendly competition can be a good motivator for some, but it can also induce negative feelings in others. Thus, competition should always be implemented carefully while considering participants’ needs and preferences. Competition is preferably introduced as a friendly strife between teams rather than between individuals, or to achieve a certain goal rather than battle against each other.

### Additional suggestions for implementation

Attention should be payed to safety and fall risk during the walk. For example, by (a) ensuring that the cognitive tasks are not too challenging and thereby take away from the safety of walking, (b) using safe walking trails, (c) providing general instructions to improve safety, (d) conducting a fall-risk screening of each participant beforehand, and (e) paying extra attention for those at higher risk of falling. In addition, it is recommended to keep the walks fun, and not too serious, especially when competition is included.

### Strengths and limitations

The main strengths of this study are that (a) the input from different angles throughout the co-design process resulted in the unique combination of experts’ and end users’ opinions, (b) each respondent could express their views anonymously, while ultimately providing information for an entire group, and (c) that no geographical constraints on the selection of experts was present and all Dutch-speaking end users could participate due to the online format of this study. However, recruiting for the survey took place online and through (walking) organisations for older adults which might have led to the overrepresentation of high-functioning, physically active respondents and the underrepresentation of those from lower socioeconomic groups and aged 85 years or above. Second, there were low response rates in the Delphi study and high drop-out rates in the survey. This could be explained by the high levels of commitment and time investment that were required, i.e. experts were asked to participate in several rounds and end users had to fill in a relatively long survey. Also, the coincidence of the final Delphi round with the start of the Covid-19 pandemic may have negatively affected response rate for the Delphi study, and the lack of digital skills in older adults may have positively affected drop-out rates in the online survey for end users. Third, an online survey is not the most optimal methodology to obtain in-depth insights in people’s opinions and experiences. However, in the light of the pandemic, it was the best way to safely gather input from a large group of end users.

### Future directions

The next step is to translate the concept that resulted from this research into an actual cognitively enriched walking program for older adults aged 65 and above. An RCT will be performed to evaluate the enjoyability and feasibility of this program and its effectiveness to improve cognitive function and physical activity. Additionally, some other directions for future research can be highlighted. First, future work could focus on the involvement of coaches in a PA + CA-program. For example, by examining how the additional burden placed on these coaches can be minimized. In particular, it could be interesting to see how coaches can be optimally prepared to appropriately integrate cognitive tasks during a physical activity such as walking (e.g. by means of training sessions and well-prepared materials, including differentiation options, to guide each walk). Second, alternatives for group walking could be considered, especially in times of Covid-19. For instance, providing cognitively enriched walks individually (e.g. by audio messaging or cueing of questions while walking past certain landmarks on a predefined walking route). Finally, future research could investigate which adaptations are needed to make the program acceptable and effective for older adults with physical and cognitive challenges such as mild cognitive impairments or dementia (e.g. performing cognitive tasks during a break).

## Conclusions

This study conceptualised a real-life, cognitively enriched walking program for older adults, using a co-design process involving academic experts and end users. Clear recommendations are provided to guide the next steps in the program development and evaluation. This includes offering a range of cognitive tasks with the opportunity for each task to variate, differentiate and increase difficulty when needed. In addition, the tasks should be integrated in the walk, the walk should be divided into three parts (warming up, cognitive tasks, free walking) and the program should be performed at least twice a week. The next step in the program development is to evaluate its effectiveness and feasibility in an RCT.

## Supplementary Information

**Additional file 1.** Delphi Round 1 – Questions.

**Additional file 2.** Delphi Round 2 – Questions.

**Additional file 3.** Delphi Round 3 – Questions.

**Additional file 4.** Description of the (groups of) cognitive tasks presented in the survey.

**Additional file 5.** Proposed tasks that were found unsuitable by the experts (due to feasibility reasons) to implement in a real-life walking program.

**Additional file 6.** Matrix 1 – Walking un-related tasks.

**Additional file 7.** Matrix 2 – Walking-related tasks.

**Additional file 8.** Matrix 3 – Walking as cognitive challenging task.

**Additional file 9.** Flow chart of end users in the survey.

## Data Availability

The datasets used and/or analysed during the current study are available from the corresponding author on reasonable request.

## Abbreviations

PA: Physical Activity
CA: Cognitive Activity
PA + CA: Combined Physical and Cognitive Activity
