# Supplementary material for: Conceptualization of a cognitively enriched walking program for older adults: a co-design study with experts and end users
Source: BMC Geriatr. 2022 Mar 1;22:167. doi: 10.1186/s12877-022-02823-z (PMC8885319; doi:10.1186/s12877-022-02823-z)
Supplement: Supplementary file 1 — Additional file 1. Delphi Round 1 – Questions. [file 12877_2022_2823_MOESM1_ESM.docx]

**Additional File 1. Delphi Round 1** **– Questions**

Type of cognitive functions to be trained

1. Which specific types of cognitive functions should be targeted in order to optimally boost neuroplasticity in healthy older adults (65+) during a 30 min walking program? (e.g. reaction speed, logical reasoning, coordination, memory…)
2. Could you briefly indicate why exactly these should be targeted?

Characteristics tasks

1. In order to improve cognitive function, how often should a certain task be performed to stimulate that aspect of cognitive function?
2. *During a 30-min walk:*
3. *During the week:*
4. Should the program focus on only specific cognitive functions or should the provided tasks target several cognitive functions at once? Please briefly indicate why as well.

Examples

1. Please give at least 3 concrete examples of cognitive tasks that could be performed during an organised group walk of 30 minutes and that, in your opinion, are useful to boost neuroplasticity and fun.
2. *Example 1:*
3. *Example 2:*
4. *Example 3:*
5. *Additional example/info:*
6. *Additional example/info:*
7. In addition, explain why these potential tasks are preferable to you.
