# Supplementary material for: Conceptualization of a cognitively enriched walking program for older adults: a co-design study with experts and end users
Source: BMC Geriatr. 2022 Mar 1;22:167. doi: 10.1186/s12877-022-02823-z (PMC8885319; doi:10.1186/s12877-022-02823-z)
Supplement: Supplementary file 2 — Additional file 2. Delphi Round 2 – Questions. [file 12877_2022_2823_MOESM2_ESM.docx]

**Additional File 2. Delphi Round 2 – Questions**

Type of cognitive functions to be trained

The answer options of the questions below are based on answers given by experts in round 1.

*Important: If you do not want to answer, you believe this is not your field of expertise or you do not agree with the given answer options, please select "Other" and specify your reason (e.g. “no expertise”, “I have doubts about this program”, “I think another answer is more suitable, namely____”).*

1. In your opinion, which specific types of cognitive functions should be targeted to optimally boost neuroplasticity in healthy older adults (65+) during a 30 min walking program? You can choose multiple answers, with a maximum of three.
2. *Executive functioning & Higher-order thinking*
3. *Attention*
4. *Memory & Learning*
5. *Processing speed*
6. *Visuospatial functions*
7. *Auditory functions*
8. *Language*
9. *Other (please specify or use "/")*
10. If in question 1 you answered "Memory", could you specify which memory systems should be targeted? Multiple answers are possible. [You can leave this question blank if you did not choose "Memory&Learning" in question 1]
11. *Working memory (= short-term memory): verbal/phonological loop*
12. *Working memory (= short-term memory): visuospatial*
13. *Long-term memory (= prospective memory): explicit/declarative, episodic memory (= relational memory)*
14. *Long-term memory (= prospective memory): explicit/declarative, semantic memory*
15. *Long-term memory (= prospective memory): implicit, procedural memory*
16. *Other (please specify)*
17. If in question 1 you answered "Executive functions", could you specify which type of executive functions should be targeted? Multiple answers are possible. [You can leave this question blank if you did not choose "Executive Functions" in question 1]
18. *Cognitive Inhibition/Inhibitory control: ability to stay focused despite distraction + inhibit pre-potent but inappropriate responses*
19. *Working Memory: ability to hold and manipulate information, priorities dual task and plan actions*
20. *Cognitive flexibility: ability to adjust and change attention, set-shifting as well as task switching*
21. *(Logical) Reasoning*
22. *Problem Solving*
23. *Decision Making*
24. *Planning*
25. *Other (please specify)*
26. If not already done in round 1, could you provide us with specific fun and feasible examples of a task that target the cognitive function(s) you answered in question 1? Please note that these tasks should be performed during a 30 min walk.
27. *Executive functioning & Higher-order thinking:*
28. *Attention:*
29. *Memory & Learning:*
30. *Processing speed:*
31. *Visuospatial functions:*
32. *Auditory functions:*
33. *Language:*
34. *Other (please specify cognitive function as well):*

Bonus (optional): do you have something to add to your previous answers? Remarks, explanations...?

1. In your opinion, what is the importance of the following reasons in the selection of the types of cognitive functions to target in a walking program?

|  | Not important | Slightly important | Moderately important | Important | Very important |
| --- | --- | --- | --- | --- | --- |
| Focus on those types will be optimal in terms of real-life relevance. | □ | □ | □ | □ | □ |
| Focus on those types that will produce the greatest physiological change. | □ | □ | □ | □ | □ |
| Focus on those types that will be optimal in terms of transfer of skill. | □ | □ | □ | □ | □ |

Bonus (optional): do you have something to add to your previous answers? Remarks, explanations...?

Characteristics tasks

1. In your opinion, at least how much time during the 30-min walk (minimum amount) should be allocated to the cognitive activities in order to improve cognitive function? Choose one.
2. *0-5 min*
3. *5-10 min*
4. *10-15 min*
5. *15-20 min*
6. *20-25 min*
7. *25-30 min*
8. *I would not walk and simultaneously perform cognitive activities.*
9. In your opinion, at least how often per week (minimum amount) should they follow the cognitively enriched walking program (30 min walk) in order to improve cognitive function? Choose one.
10. *1*
11. *2*
12. *3*
13. *4*
14. *5*
15. *6*
16. *7 (every day of the week)*
17. *I would not walk and simultaneously perform cognitive activities.*

Bonus (optional): do you have something to add to your previous answers? Remarks, explanations...?
