# Supplementary material for: Conceptualization of a cognitively enriched walking program for older adults: a co-design study with experts and end users
Source: BMC Geriatr. 2022 Mar 1;22:167. doi: 10.1186/s12877-022-02823-z (PMC8885319; doi:10.1186/s12877-022-02823-z)
Supplement: Supplementary file 3 — Additional file 3. Delphi Round 3 – Questions. [file 12877_2022_2823_MOESM3_ESM.docx]

**Additional File 3. Delphi Round 3 – Questions**

The final statements below are based on answers given by experts in round 1 and 2. You are asked to indicate - for the last time - your level of agreement on these statements.

*Important:*

- *Please do not hesitate to share a view that differs from the summary feedback. There is a possibility to express remarks at the bottom of the page.*
- *If you do not want to answer or you believe this is not your field of expertise, please select "Other" and specify your reason (e.g. “no expertise”) or use "/".*

1. Please indicate the degree to which you agree/disagree with the following statements:
   1. “The PA+CA program should primarily target executive functioning and higher-order thinking, as well as memory and learning."

| Strongly disagree | Disagree | Neither agree nor disagree | Agree | Strongly agree | Other |
| --- | --- | --- | --- | --- | --- |
| □ | □ | □ | □ | □ | □ namely... |

- 1. “With regard to executive functioning and higher-order thinking, the program should mostly focus on working memory, cognitive inhibition/inhibitory control, cognitive flexibility and planning.”

| Strongly disagree | Disagree | Neither agree nor disagree | Agree | Strongly agree | Other |
| --- | --- | --- | --- | --- | --- |
| □ | □ | □ | □ | □ | □ namely... |

- 1. “With regard to memory and learning, the program should mostly focus on the visuospatial and verbal/phonological loop of the working memory.”

| Strongly disagree | Disagree | Neither agree nor disagree | Agree | Strongly agree | Other |
| --- | --- | --- | --- | --- | --- |
| □ | □ | □ | □ | □ | □ namely... |

- 1. "The most important reason in selecting the targeted cognitive functions for the PA+CA-program is their real-life relevance.”

| Strongly disagree | Disagree | Neither agree nor disagree | Agree | Strongly agree | Other |
| --- | --- | --- | --- | --- | --- |
| □ | □ | □ | □ | □ | □ namely... |

- 1. "During the 30-min walk, minimum 15-20 minutes should be allocated to cognitive activities in order to improve cognitive function."

| Strongly disagree | Disagree | Neither agree nor disagree | Agree | Strongly agree | Other |
| --- | --- | --- | --- | --- | --- |
| □ | □ | □ | □ | □ | □ namely... |

- 1. "During the week, the PA+CA program should be organized at least 2-3 times in order to improve cognitive function."

| Strongly disagree | Disagree | Neither agree nor disagree | Agree | Strongly agree | Other |
| --- | --- | --- | --- | --- | --- |
| □ | □ | □ | □ | □ | □ namely... |

Bonus (optional): do you have something to add to your previous answers? Remarks, explanations...?

We have collected and categorized all the different examples of cognitive tasks that were given by the experts in rounds 1 and 2. Below you will find three major categories (the three matrices), subdivided in several subcategories (columns) with specific examples of tasks, sorted according to complexity (along y-axis). Please have a thorough look at these three matrices and answer the questions below them.

*Matrix 1*

| 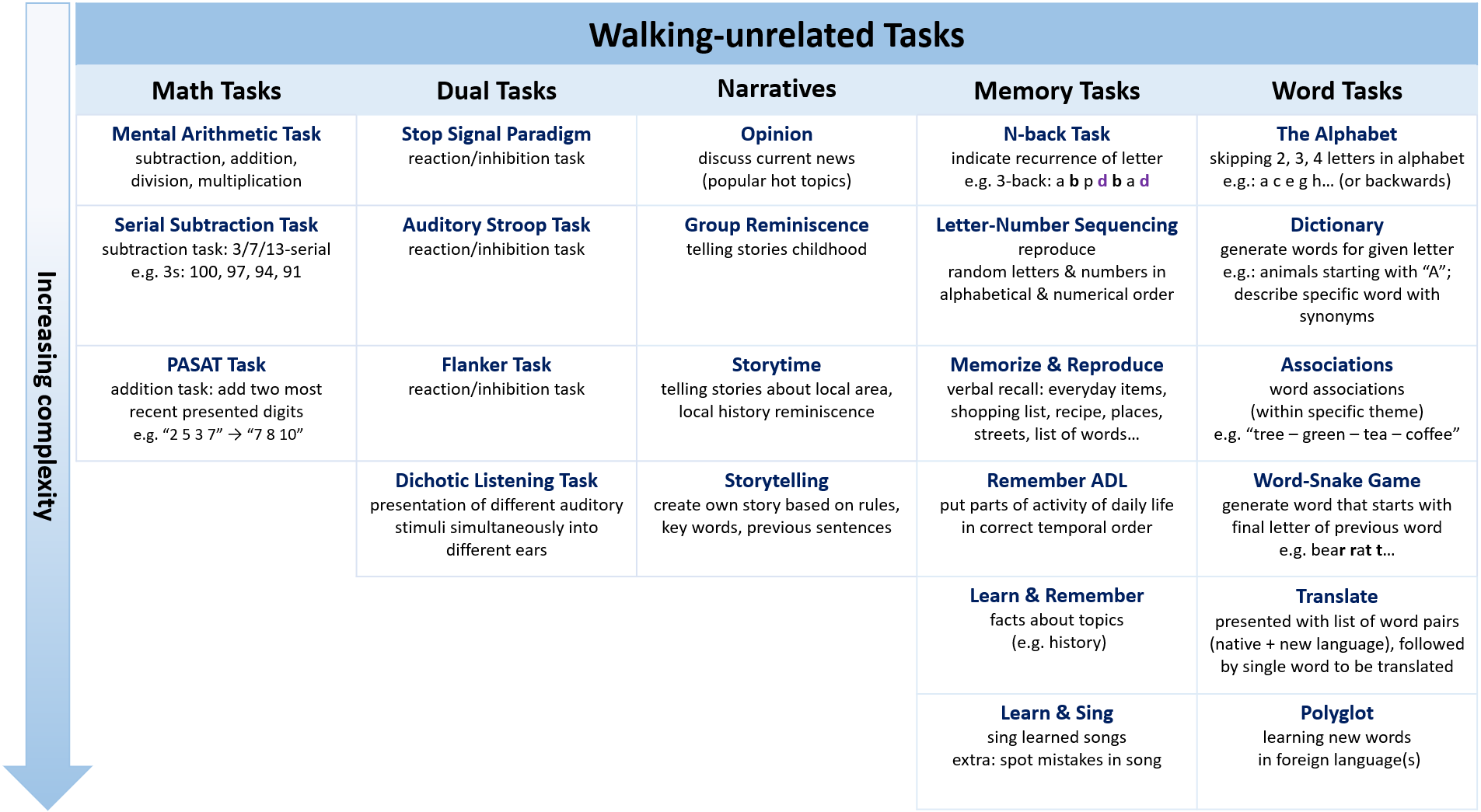  The following tasks were categorized as “Waking-unrelated Tasks”. This means they are essentially unrelated to walking; walking is not a necessary condition. In other words, you do not have to walk to take on these tasks. Nevertheless, as we aim to establish a PA+CA-program, we plan to perform/integrate these tasks during our walks.  Math Tasks   1. Mental Arithmetic Task: provide arithmetical operations to train mental calculation abilities. 2. Serial Subtraction Task: ask to subtract x (e.g. 3; 7; 13) from y (e.g. 100) and keep going (serial 3s: 100, 97, 94…). 3. PASAT Task (**P**aced **A**uditory **S**erial **A**ddition): presenting series of single digit numbers where the two most recent digits must be summed (e.g. present “2, 5, 3, 7” → “7 (= 5+2), 8 (5+3), 10 (=7+3)”).   Dual Tasks   1. Stop Signal Paradigm: present series of “Go” signals, instruct to respond quickly; however, also present “Stop” signals to which participants are instructed to inhibit their response. 2. Auditory Stroop Task: respond as quickly as possible to some perceptual feature of word (e.g., speaker gender, voice pitch, stimulus location) while ignoring semantic information. 3. Flanker Task: ask directional response to central target stimulus, while ignoring non-target stimuli presented in its immediate proximity. 4. Dichotic Listening Task: listen to different acoustic events presented to each ear simultaneously.   Narratives   1. Opinion: discuss the current news (popular hot topic) 2. Group Reminiscence: let them tell stories about their childhood; their life as a 45-year-old. 3. Storytime: let them tell stories about the local area (local history reminiscence) 4. Storytelling:    1. Create a story with the group: each person says new sentence following the previous one    2. Story needs to be developed according to rules and key words   Memory Tasks   1. N-back Task: reading list of letters, ask to indicate when letter is same as *N* letters ago (e.g. 3-back: a b p d **b** a d) 2. Letter-Number Sequencing: sequence random order of numbers & letters, say numbers in ascending order and then letters in alphabetical order. 3. Memorize & Reproduce: memorize list of items and reproduce them as good as possible 4. Remember ADL: sort sketches of an activity of daily living, provided on sheet of papers, in correct temporal order 5. Learn & Remember: learn specific facts about topics such as history and try to remember them 6. Learn & Sing: sing learned songs while walking (extra: spot lyrical/rhythmic mistakes in played songs)   Word Tasks   1. The Alphabet: going (backwards) through the alphabet, skipping e.g. 1 letter each time 2. Dictionary:    1. Verbal fluency, generate words (within a category such as animals) from given initial letter (e.g. a,f,s,c,l).    2. Describe things as elaborated as possible or use different words for the same things 3. Associations:    1. Word associations game (“blue – sky – airplane – pollution – protests”)    2. Find words within a specific theme/category (e.g., fruits and vegetables) 4. Word-Snake Game: generate words that start with final letter of previous word (e.g., bea**r – r**a**t – t**…) 5. Translate: auditory presented list of word pairs (native and new language), followed by a single word that has to be translated. 6. Polyglot: learn new words in a foreign language |
| --- |

In your opinion, are all tasks presented in the matrix above suited as **effective** cognitive activities? Performing them during an organised group walk of 30 minutes should eventually help the older adults at maintaining and improving their cognitive capabilities.

- *I do not wish to answer*
- *Yes, all presented tasks are suited as effective cognitive activities*
- *No (please specify which task(s) are not suited):*

In your opinion, are all tasks presented in the matrix above suited as **feasible** cognitive activities according to you? Older adults (healthy, 65+ years old) should be able to do them during an outdoor walk in group, under the supervision of a coach.

- *I do not wish to answer*
- *Yes, all presented tasks are suited as feasible cognitive activities*
- *No (please specify which task(s) are not suited):*

Do you agree with our overall classification of the tasks as “Walking-unrelated Tasks”? This means they are essentially unrelated to walking; walking is not a necessary condition. In other words, you do not have to walk to take on these tasks. Nevertheless, as we aim to establish a PA+CA-program, we plan to perform/integrate these tasks during our walks.

- - *I do not wish to answer*
  - *Yes, all presented tasks are accurately classified as “Walking-unrelated Tasks”*
  - *No (please specify which task(s) are wrongly classified):*

Do you agree with our classification of the tasks within the subcategories “Math Tasks – Dual Tasks – Narratives – Memory Tasks – Word Tasks”?

- - *I do not wish to answer*
  - *Yes, all presented tasks are accurately classified within each subcategory*
  - *No (please specify which task(s) are wrongly classified):*

The tasks are ranked within each subcategory (column) according to their **complexity level** (see arrow on the left). This is based on our estimation of how cognitive challenging each task is at baseline level and on how much differentiation and variation is possible within each task (suitability to scale it up). Do you agree on how all tasks are ranked according to complexity (within each subcategory)? Please note that each task can be differentiated and can have increasing complexity levels, but consider for now a basic level.

- - *I do not wish to answer*
  - *Yes, all presented tasks are accurately ranked according to complexity*
  - *No (please specify which task(s) are wrongly ranked):*

Bonus (optional): Some of the experts suggested a few tasks that we would value your thoughts and opinion on. How would you implement the following tasks in a real-life walking program? In other words, which adaptations could be made to incorporate these tasks in a walking and outdoor environment?

- *Auditory Stroop Task:*
- *Flanker Task:*
- *Dichotic Listening Task:*

Bonus (optional): do you have something to add to your previous answers? Remarks, explanations...?

*Matrix 2*

| 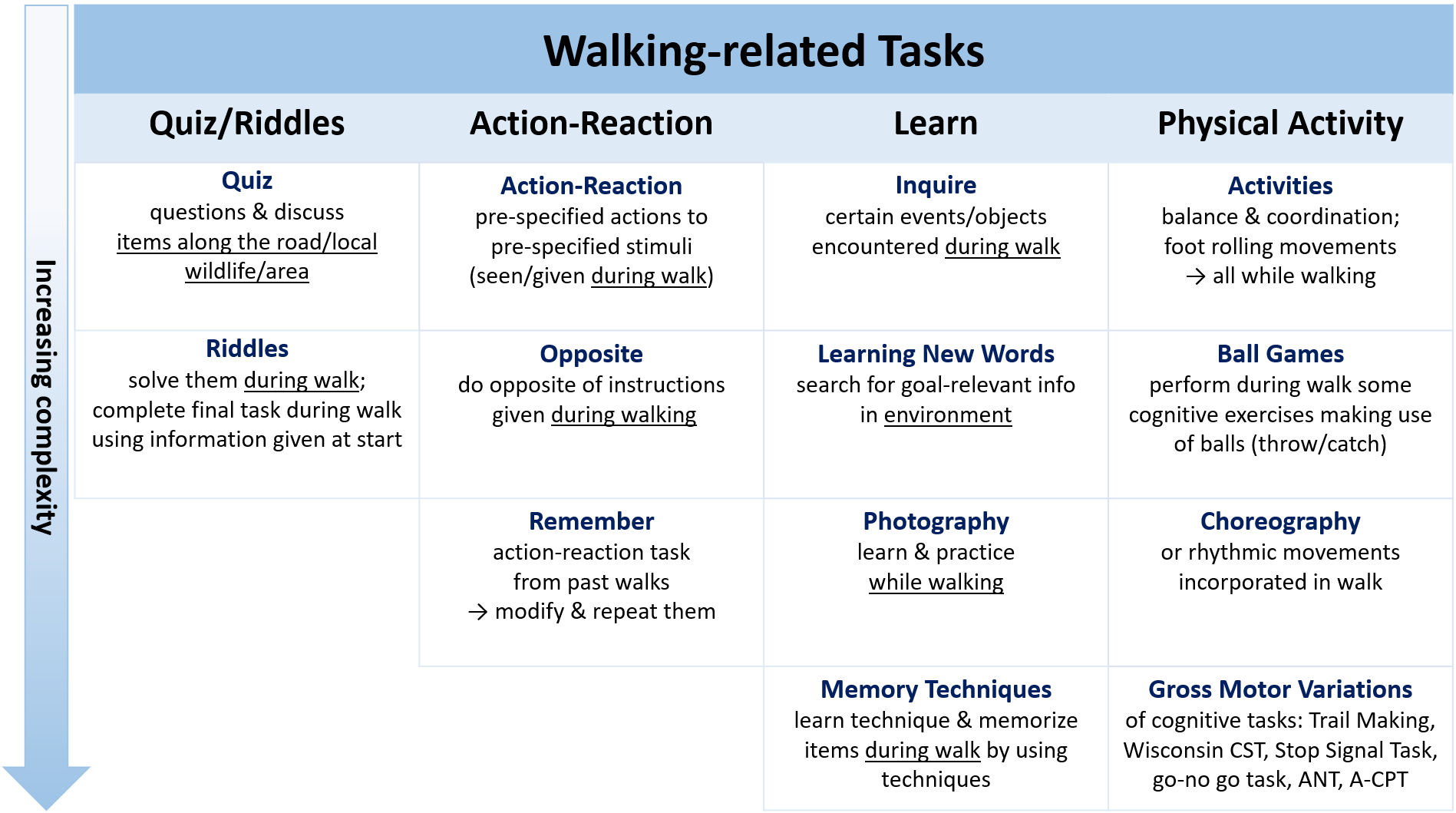  The following tasks were categorized as “Walking-related Tasks”. This means they are related to walking, namely you do have to walk to take on these tasks. The walking even becomes a fundamental part of the tasks – a necessary condition – and is of added value. Therefore, leaving out the walking part is possible, but will fundamentally change the original, specific intent of the tasks.  Quiz/Riddles   1. Quiz:    1. Hold a quiz on local wildlife/area, neighbourhood (let them discuss to find correct answer)    2. Every 5 minutes 1-2 questions about items along the road 2. Riddles:    1. Present several riddles they need to solve during walking (problem-thinking).    2. Completing a final task using information given at the start of the walk   Action-Reaction   1. Action-Reaction: pre-specified reactions on pre-specified stimuli (cues, signs) 2. Opposite: do opposite of instruction (inhibit initial reaction) 3. Remember: ask to remember an action-reaction task of the past walks, modify & repeat them   Learn   1. Inquire: inform participants about objects/events encountered during the walk 2. Learning New Words: search for goal-relevant information in environment to learn new words 3. Photography: learn the technique of making photographs and practice during the walk 4. Memory Techniques: learn and practice memory techniques (e.g. mnemonics, Loci)   Physical Activity   1. Activities:    1. Do some balance & coordination exercises    2. Focus on foot rolling movements while naming items in area 2. Ball Games: throw balls to each other (specific order, work with different colours, different rules) 3. Choreography: learn a choreography while walking, do rhythmic movements during walk 4. Gross Motor Variations of specific cognitive tasks:    1. Trail Making: draw lines to connect circled numbers in a numerical sequence/to connect circled numbers and letters in an alternating numeric and alphabetic sequence (as quick and accurate as possible)    2. Wisconsin Card Sorting Task: sort cards according to different rules (colour, form…)    3. Go-no go Task: One of four shapes (circle, square, triangle, or diamond) is randomly designated as a “nontarget” for each participant. Participants are instructed to respond as fast and as accurately as possible anytime a target shape appears (i.e., go trial) but to withhold their response whenever the nontarget shape appears (i.e., no-go trial).    4. Stop Signal Task: present series of “Go” stimuli, instruct to respond quickly; however, also present “stop” signals to which participants are instructed to inhibit their response.    5. Attention Network Task: determine as fast and accurately as possible direction of a central arrow (target) located in middle of horizontal line projected either at top or at bottom of screen.    6. Auditory Continuous Performance Task: presented with a repetitive, boring task; must maintain focus over a period of time in order to respond to targets or inhibit response to foils |
| --- |

In your opinion, are all tasks presented in the matrix above suited as **effective** cognitive activities? Performing them during an organised group walk of 30 minutes should eventually help the older adults at maintaining and improving their cognitive capabilities.

- *I do not wish to answer*
- *Yes, all presented tasks are suited as effective cognitive activities*
- *No (please specify which task(s) are not suited):*

In your opinion, are all tasks presented in the matrix above suited as **feasible** cognitive activities according to you? Older adults (healthy, 65+ years old) should be able to do them during an outdoor walk in group, under the supervision of a coach.

- *I do not wish to answer*
- *Yes, all presented tasks are suited as feasible cognitive activities*
- *No (please specify which task(s) are not suited):*

Do you agree with our overall classification of the tasks as “Walking-related Tasks”? This means they are related to walking, namely you do have to walk to take on these tasks. The walking even becomes a fundamental part of the tasks – a necessary condition – and is of added value. Therefore, leaving out the walking part is possible, but will fundamentally change the original, specific intent of the tasks.

- - *I do not wish to answer*
  - *Yes, all presented tasks are accurately classified as “Walking-related Tasks”*
  - *No (please specify which task(s) are wrongly classified):*

Do you agree with our classification of the tasks within the subcategories “Quiz/Riddles – Action-Reaction – Learn – Physical Activity”?

- - *I do not wish to answer*
  - *Yes, all presented tasks are accurately classified within each subcategory*
  - *No (please specify which task(s) are wrongly classified):*

The tasks are ranked within each subcategory (column) according to their **complexity level** (see arrow on the left). This is based on our estimation of how cognitive challenging each task is at baseline level and on how much differentiation and variation is possible within each task (suitability to scale it up). Do you agree on how all tasks are ranked according to complexity (within each subcategory)? Please note that each task can be differentiated and can have increasing complexity levels, but consider for now a basic level.

- - *I do not wish to answer*
  - *Yes, all presented tasks are accurately ranked according to complexity*
  - *No (please specify which task(s) are wrongly ranked):*

Bonus (optional): Some of the experts suggested a few tasks that we would value your thoughts and opinion on. How would you implement the following tasks in a real-life walking program? In other words, which adaptations could be made to incorporate these tasks in a walking and outdoor environment? It was suggested to make gross motor variations of it, but how would you explicitly do this?

- *Wisconsin Card Sorting Task:*
- *Attention Network Task:*
- *Auditory Continuous Performance Task:*

Bonus (optional): do you have something to add to your previous answers? Remarks, explanations...?

*Matrix 3*

| 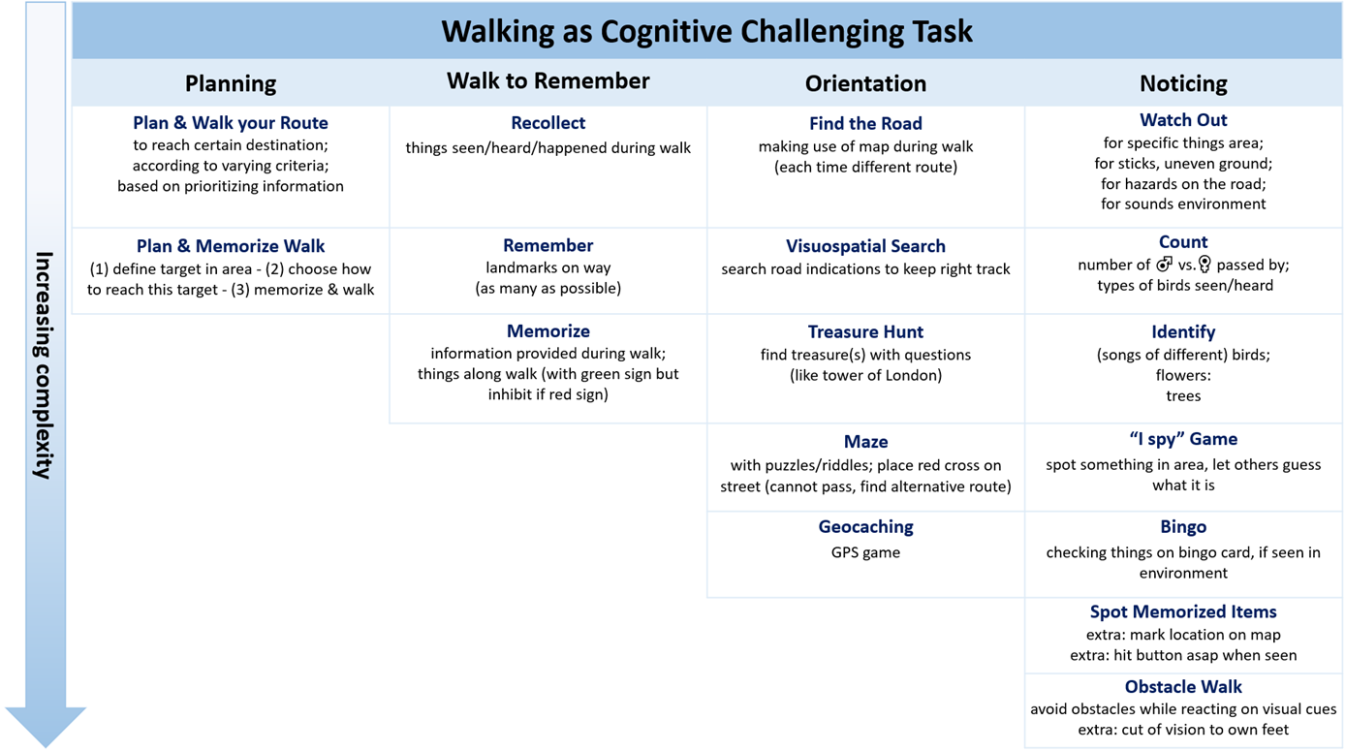  The following tasks were categorized as “Walking as Cognitive Challenging Task”. This means that the walk itself becomes the cognitive challenge. Walking is no longer a fundamental part of the task, it becomes the task. Walking and the task are inextricably linked. It is essential to walk in order to take on the tasks.  Planning   1. Plan your Route:    1. To reach a certain destination    2. According to varying criteria    3. Based on prioritizing information 2. Plan & Memorize Walk: (1) define a specific target in town - (2) choose how you will reach this target, use a map to plan your route - (3) memorize your route - (4) walk towards the target   Walk to Remember   1. Recollect: at end of walk things that happened or things they saw/heard during walk 2. Remember: as many landmarks as possible on the way 3. Memorize:    1. Information provided during walk    2. Things along the walk that have a green sign, but inhibit when it has a red sign   Orientation   1. Find the Road: by making use of a map during walk (each walk must have different route) 2. Visuospatial Search: search for road indications to keep the right track 3. Treasure Hunt: find treasure(s) along way by answering questions/riddles (e.g. tower of London) 4. Maze:    1. Make the walk a maze by giving them puzzles/riddles that need to be solved in order to find the correct way out    2. Place red crosses on streets they cannot pass, resulting in searching for alternative routes 5. Geocaching: find specific locations by using GPS coordinates   Noticing   1. Watch Out:    1. for specific things in the area    2. for sticks, uneven ground    3. for hazards on the road    4. for specific sounds in the environment 2. Count:    1. the number of males/females passed on the walk    2. the types of birds seen/heard during the walk    3. tones/tapping produced by the coach/others participants 3. Identify:    1. The song of different birds    2. Specific flowers    3. Trees 4. “I Spy” Game: spot something in area, let others guess what it is by describing it (e.g., “it’s green”) 5. Bingo: checking things off on a bingo card, if seen in environment. All found? BINGO! 6. Spot Memorized Items: Memorize beforehand a list of items you need to spot during the walk. Keep track on how many items you saw and try to indicate on a map where you have seen it. 7. Obstacle Walk:    1. Walk in a trail with obstacles, do not step on obstacles while reacting on visual cues (e.g. the trainer shows numbers)    2. Walk in a trail with obstacles, focus on foot rolling movements and react on visual or auditory cues    3. Cut of the vision to the own feet by using sight screens while avoiding obstacles |
| --- |

In your opinion, are all tasks presented in the matrix above suited as **effective** cognitive activities? Performing them during an organised group walk of 30 minutes should eventually help the older adults at maintaining and improving their cognitive capabilities.

- *I do not wish to answer*
- *Yes, all presented tasks are suited as effective cognitive activities*
- *No (please specify which task(s) are not suited):*

In your opinion, are all tasks presented in the matrix above suited as **feasible** cognitive activities according to you? Older adults (healthy, 65+ years old) should be able to do them during an outdoor walk in group, under the supervision of a coach.

- *I do not wish to answer*
- *Yes, all presented tasks are suited as feasible cognitive activities*
- *No (please specify which task(s) are not suited):*

Do you agree with our overall classification of the tasks as “Walking as Cognitive Challenging Task”? This means that the walk itself becomes the cognitive challenge. Walking is no longer a fundamental part of the task, it becomes the task. Walking and the task are inextricably linked. It is essential to walk in order to take on the tasks.

- - *I do not wish to answer*
  - *Yes, all presented tasks are accurately classified as “Walking as Cognitive Challenging Task”*
  - *No (please specify which task(s) are wrongly classified):*

Do you agree with our classification of the tasks within the subcategories “Planning – Walk to Remember – Orientation – Noticing”?

- - *I do not wish to answer*
  - *Yes, all presented tasks are accurately classified within each subcategory*
  - *No (please specify which task(s) are wrongly classified):*

The tasks are ranked within each subcategory (column) according to their **complexity level** (see arrow on the left). This is based on our estimation of how cognitive challenging each task is at baseline level and on how much differentiation and variation is possible within each task (suitability to scale it up). Do you agree on how all tasks are ranked according to complexity (within each subcategory)? Please note that each task can be differentiated and can have increasing complexity levels, but consider for now a basic level.

- - *I do not wish to answer*
  - *Yes, all presented tasks are ranked accurately according to complexity*
  - *No (please specify which task(s) are wrongly ranked):*

Bonus (optional): do you have something to add to your previous answers? Remarks, explanations...?
