# Supplementary material for: Conceptualization of a cognitively enriched walking program for older adults: a co-design study with experts and end users
Source: BMC Geriatr. 2022 Mar 1;22:167. doi: 10.1186/s12877-022-02823-z (PMC8885319; doi:10.1186/s12877-022-02823-z)
Supplement: Supplementary file 4 — Additional file 4. Description of the (groups of) cognitive tasks presented in the survey. [file 12877_2022_2823_MOESM4_ESM.docx]

**Additional File 4. Description of the (groups of) cognitive tasks presented in the survey.**

1. **Facts and titbits**: Walkers tell each other a fact or titbit about something they know (e.g. nature, history, buildings, a funny anecdote) and try to remember what the others tell. Afterwards, everyone is allowed to pose one quiz question about the fact that he or she told.
2. **Quest with environmental clues**: A quest in which participants can find the right route by searching for clues in their environment (e.g. follow the arrows). Clues can also be given at the start of the walk (e.g. a photo of something on the route), walkers then have to find the place where the photo was taken.
3. **Awareness**: Walkers try to become aware and make others aware of certain things. For instance, themselves (e.g. their own movements, breathing), their surroundings (e.g. remarkable noises, objects, obstacles). Walkers are encouraged to count remarkable things and to try to remember these things at the end of the walk.
4. **Spotted**: Walkers try to spot certain objects (e.g. a bird, a mushroom, a church). They can try to find it as fast as possible or as many as possible, they can call ‘bingo’ when all objects from a pre-arranged list are found, they can try to take a picture of every spotted item or they can use a road map to indicate where the item was spotted.
5. **Opinions**: Walkers share their opinion about an imposed theme (e.g. hot topics in the news or historical events).
6. **Notice and remember symbols**: Walkers search for pre-applied symbols on the walk and try to remember which symbol was found on which object.
7. **Quiz**: Walkers answer quiz questions about things they walked past, local fauna and flora, buildings and monuments, local history, …
8. **Plan the route**: Walkers plan the walking route themselves and draw it on a road map. They can try to remember the planned route or use the map while walking. Rules can be agreed upon to make it more challenging (e.g. the route must pass past a bakery or certain roads cannot be used).
9. **Quest**: A quest in which the walkers solve problems, riddles and quiz questions in order to gain information about the route they have to walk.
10. **Hidden** **word**: One walker describes a word (e.g. object, place, event, well-known person) while the other walkers try to guess the word.
11. **Words** **starting** **with** **a particular letter**: Walkers call words in a pre-arranged theme (e.g. animals, vegetables, cities) that start with a predetermined letter.
12. **Problem solving**: Walkers try to solve riddles and other problems.
13. **Word associations**: Walkers make a chain of word associations by calling the first word that comes to mind (e.g. blue – air – plane – pollution).
14. **Remember the route**: Walkers try to remember the route while they are walking. Afterwards, they try to draw the route on a map.
15. **I spy**: Walkers play the well-known game ‘I spy with my little eye’.
16. **A new language**: Walkers learn a few words or sentences in a foreign language.
17. **Buzz it**: Walkers come up with an answer to a question as quickly as possible. These questions require inventiveness rather than knowledge (e.g. What do you give as a birth gift? Where should you definitely not put your finger in?).
18. **Story telling**: Walkers create a story by adding a sentence to the sentence of the previous person. Rules can be agreed upon to make it more challenging (e.g. certain words or rhyme schemes must be used).
19. **Geocaching**: Walkers use the geocaching-app to play the well-known game ‘geocaching’.
20. **Serial subtraction task**: Walkers perform the serial subtraction task, in which they count down from 100 in steps of 3, 7 or 13.
21. **Memory techniques**: Walkers learn how to use certain memory techniques (e.g. Loci-method).
22. **Music**: Walkers learn a song and sing it, try to sing along with the played music, try to continue singing in the wright rhythm when the music stops or try to guess the title and artist of a played song.
23. **Obstacle** **walk**: Participants walk a parkours with obstacles.
24. **Order of daily activities**: All walkers receive a part of an activity (e.g. repair a bicycle tire, maintain a vegetable garden from sowing to harvesting). They try to put all parts in the right order by discussing and portraying the activity.
25. **Mental arithmetic**: Walkers solve mental arithmetic tasks (e.g. addition tasks, subtraction tasks, multiplication tasks, division tasks or the PASAT task).
26. **The alphabet**: Walkers recite the alphabet in different ways (e.g. by starting with a letter other than the a, by skipping one or two letters each time, by saying it backwards, or by alternating the letters with numbers).
27. **Choreography**: Walkers invent special ways to walk and cast them into some kind of choreography (e.g. take five big steps, then five small steps on your toes, jump backwards with both feet together, clap in your hands and start all over).
28. **Immediate** **recall**: Walkers perform an immediate recall test with a list of numbers, letters or words. The items can be recalled in a random order, in the same order, backwards or in a logical (e.g. alphabetical) order.
29. **N-back**: Walkers perform an N-back task (react if a stimulus matches with the stimulus n trials before) with a list of numbers, letters or words.
30. **List learning**: Walkers perform a list learning task in which they memorize relevant information (e.g. phone numbers, shopping lists, addresses) for a longer period of time.
31. **Stimulus-response**: Walkers perform certain stimulus-response tasks (e.g. jump when you hear a bird, clap in your hands when you see a yellow car). Walkers can agree upon more than one stimulus-response task, they can add stop signals (e.g. clap in your hands when you see a yellow car, except when it has a foreign number plate) or they can play the well-known game ‘Simon says’.
32. **Ball-games**: Walkers throw soft balls at each other. They can agree upon rules about to whom they have to throw which ball or how a certain ball has to be thrown.
