# Supplementary material for: Conceptualization of a cognitively enriched walking program for older adults: a co-design study with experts and end users
Source: BMC Geriatr. 2022 Mar 1;22:167. doi: 10.1186/s12877-022-02823-z (PMC8885319; doi:10.1186/s12877-022-02823-z)
Supplement: Supplementary file 5 — Additional file 5. Proposed tasks that were found unsuitable by the experts (due to feasibility reasons) to implement in a real-life walking program. [file 12877_2022_2823_MOESM5_ESM.docx]

**Additional File 5. Proposed tasks that were found unsuitable by the experts (due to feasibility reasons) to implement in a real-life walking program.**

| **Wisconsin Card Sorting Task** | “sort cards according to different rules (color, form…)” |
| --- | --- |
| **Flanker Task** | “ask directional response to central target stimulus, while ignoring non-target stimuli presented in its immediate proximity” |
| **Attention Network Task** | “determine as fast and accurately as possible direction of a central arrow (target) located in middle of horizontal line projected either at top or at bottom of screen” |
| **Auditory Continuous Performance Task** | “presented with a repetitive, boring task; must maintain focus over a period of time in order to respond to targets or inhibit response to foils” |
