# Supplementary material for: Conceptualization of a cognitively enriched walking program for older adults: a co-design study with experts and end users
Source: BMC Geriatr. 2022 Mar 1;22:167. doi: 10.1186/s12877-022-02823-z (PMC8885319; doi:10.1186/s12877-022-02823-z)
Supplement: Supplementary file 6 — Additional file 6. Matrix 1 – Walking un-related tasks. [file 12877_2022_2823_MOESM6_ESM.docx]

**Additional File 6. Matrix 1 – Walking un-related tasks ^a^**

| **Math Tasks** | **Inhibition Tasks** | **Narratives** | **Memory Tasks** | **Word Tasks** |
| --- | --- | --- | --- | --- |
| **Serial Subtraction Task**  *subtraction task: 3/7/13-serial*  *e.g. 3s: 100, 97, 94, 91* | **Stop Signal Paradigm**  *reaction/inhibition task* | **Group *Reminiscence***  *telling stories childhood* | **Letter-Number Sequencing**  *reproduce random letters & numbers in alphabetical & numerical order* | **The Alphabet**  *skipping 2, 3, 4 letters in alphabet*  *e.g.: a c e g h… (or backwards)* |
| **Mental Arithmetic Task**  *subtraction, addition,*  *division, multiplication* | **Stroop Task**  *reaction/inhibition task* | **Opinion**  *discuss current news*  *(popular hot topics)* | **N-back Task**  *indicate recurrence of letter*  *e.g. 3-back: a* ***b*** *p* ***d*** ***b*** *a* ***d*** | **Dictionary**  *generate words for given letter*  *e.g.: animals starting with “A”; describe specific word with synonyms* |
| **PASAT Task**  *addition task: add two most recent presented digits*  *e.g. “2 5 3 7” → “7 8 10”* | ***Dichotic Listening Task***  *presentation of different auditory stimuli simultaneously into different ears* | **Storytime**  *telling stories about local area, local history reminiscence* | **Remember ADL**  *put parts of activity of daily life*  *in correct temporal order* | **Associations**  *word associations*  *(within specific theme)*  *e.g. “tree – green – tea – coffee”* |
|  | | **Storytelling**  *create own story based on rules, key words, previous sentences* | **Learn & Remember**  *facts about topics*  *(e.g. history)* | **Word-Snake Game**  *generate word that starts with final letter of previous word*  *e.g. bea****r r****a****t*** ***t****…* |
|  |  |  | **Learn & Sing**  *sing learned songs*  *extra: spot mistakes in song* | **Translate**  *presented with list of word pairs (native + new language), followed by single word to be translated* |
|  |  |  | **Memorize & Reproduce**  *verbal recall: everyday items, shopping list, recipe, places, streets, list of words…* | **Polyglot**  *learning new words in foreign language(s)* |

*Notes.* The tasks are ranked within each subcategory (column) according to their complexity level; PASAT = Paced Auditory Serial Addition Test.

^a^ This means they are essentially unrelated to walking; walking is not a necessary condition. In other words, you do not have to walk to take on these tasks. Nevertheless, as we aim to establish a PA+CA-program, we plan to perform/integrate these tasks during our walks.
