# Supplementary material for: Conceptualization of a cognitively enriched walking program for older adults: a co-design study with experts and end users
Source: BMC Geriatr. 2022 Mar 1;22:167. doi: 10.1186/s12877-022-02823-z (PMC8885319; doi:10.1186/s12877-022-02823-z)
Supplement: Supplementary file 7 — Additional file 7. Matrix 2 – Walking-related tasks. [file 12877_2022_2823_MOESM7_ESM.docx]

**Additional File 7. Matrix 2 – Walking-related tasks ^a^**

| **Quiz/Riddles** | **Action-Reaction** | **Learn** | **Physical Activity** |
| --- | --- | --- | --- |
| **Quiz**  *questions & discuss*  *items along the road/local wildlife/area* | **Action-Reaction**  *pre-specified actions to*  *pre-specified stimuli*  *(seen/given during walk)* | **Inquire**  *certain events/objects encountered during walk* | **Activities**  *balance & coordination;*  *foot rolling movements*  *→ all while walking* |
| **Riddles**  *solve them during walk;*  *complete final task during walk using information given at start* | **Opposite**  *do opposite of instructions*  *given during walking* | **Learning New Words**  *search for goal-relevant info*  *in environment* | **Choreography**  *or rhythmic movements incorporated in walk* |
|  | **Remember**  *action-reaction task*  *from past walks*  *→ modify & repeat them* | **Photography**  *learn & practice*  *while walking* | **Ball Games**  *perform during walk some cognitive exercises making use of balls (throw/catch)* |
|  | | **Memory Techniques**  *learn technique & memorize items during walk by using techniques* | **Gross Motor Variations**  *of cognitive tasks: Trail Making, Stop Signal Task, go-no go task* |

*Note.* The tasks are ranked within each subcategory (column) according to their complexity level.

^a^ This means they are related to walking, namely you do have to walk to take on these tasks. The walking even becomes a fundamental part of the tasks – a necessary condition – and is of added value. Therefore, leaving out the walking part is possible, but will fundamentally change the original, specific intent of the tasks.
