# Supplementary material for: Conceptualization of a cognitively enriched walking program for older adults: a co-design study with experts and end users
Source: BMC Geriatr. 2022 Mar 1;22:167. doi: 10.1186/s12877-022-02823-z (PMC8885319; doi:10.1186/s12877-022-02823-z)
Supplement: Supplementary file 8 — Additional file 8. Matrix 3 – Walking as cognitive challenging task. [file 12877_2022_2823_MOESM8_ESM.docx]

**Additional File 8. Matrix 3 – Walking as cognitive challenging task ^a^**

| **Planning** | **Walk to Remember** | **Orientation** | **Noticing** |
| --- | --- | --- | --- |
| **Plan & Walk your Route**  *to reach certain destination;*  *according to varying criteria;*  *based on prioritizing information* | **Recollect**  *things* *seen/heard/happened during walk* | **Find the Road**  *making use of map during walk*  *(each time different route)* | **Watch Out**  *for specific things area;*  *for sticks, uneven ground;*  *for hazards on the road;*  *for sounds environment* |
| **Plan & Memorize Walk**  *(1) define target in area - (2) choose how to reach this target - (3) memorize & walk* | **Remember**  *landmarks on way*  *(as many as possible)* | **Visuospatial Search**  *search road indications to keep right track* | **Count**  *number of ♂ vs.♀ passed by;*  *types of birds seen/heard* |
|  | **Memorize**  *information provided during walk;*  *things along walk (with green sign but inhibit if red sign)* | **Treasure Hunt**  *find treasure(s) with questions*  *(like tower of London)* | **Identify**  *(songs of different) birds;*  *flowers:*  *trees* |
|  | | **Maze**  *with puzzles/riddles; place red cross on street (cannot pass, find alternative route)* | **“I spy” Game**  *spot something in area, let others guess what it is* |
|  | | **Geocaching**  *GPS game* | **Bingo**  *checking things on bingo card, if seen in environment* |
|  | |  | **Spot Memorized Items**  *extra: mark location on map*  *extra: hit button asap when seen* |
|  | |  | **Obstacle Walk**  *avoid obstacles while reacting on visual cues*  *extra: cut of vision to own feet* |

*Note.* The tasks are ranked within each subcategory (column) according to their complexity level.

^a^ This means that the walk itself becomes the cognitive challenge. Walking is no longer a fundamental part of the task, it becomes the task. Walking and the task are inextricably linked. It is essential to walk in order to take on the tasks.
