# Supplementary figures and images for: Conceptualization of a cognitively enriched walking program for older adults: a co-design study with experts and end users
Source: BMC Geriatr. 2022 Mar 1;22:167. doi: 10.1186/s12877-022-02823-z (PMC8885319; doi:10.1186/s12877-022-02823-z)

**Additional File 9. Flow chart of end users in the survey.**


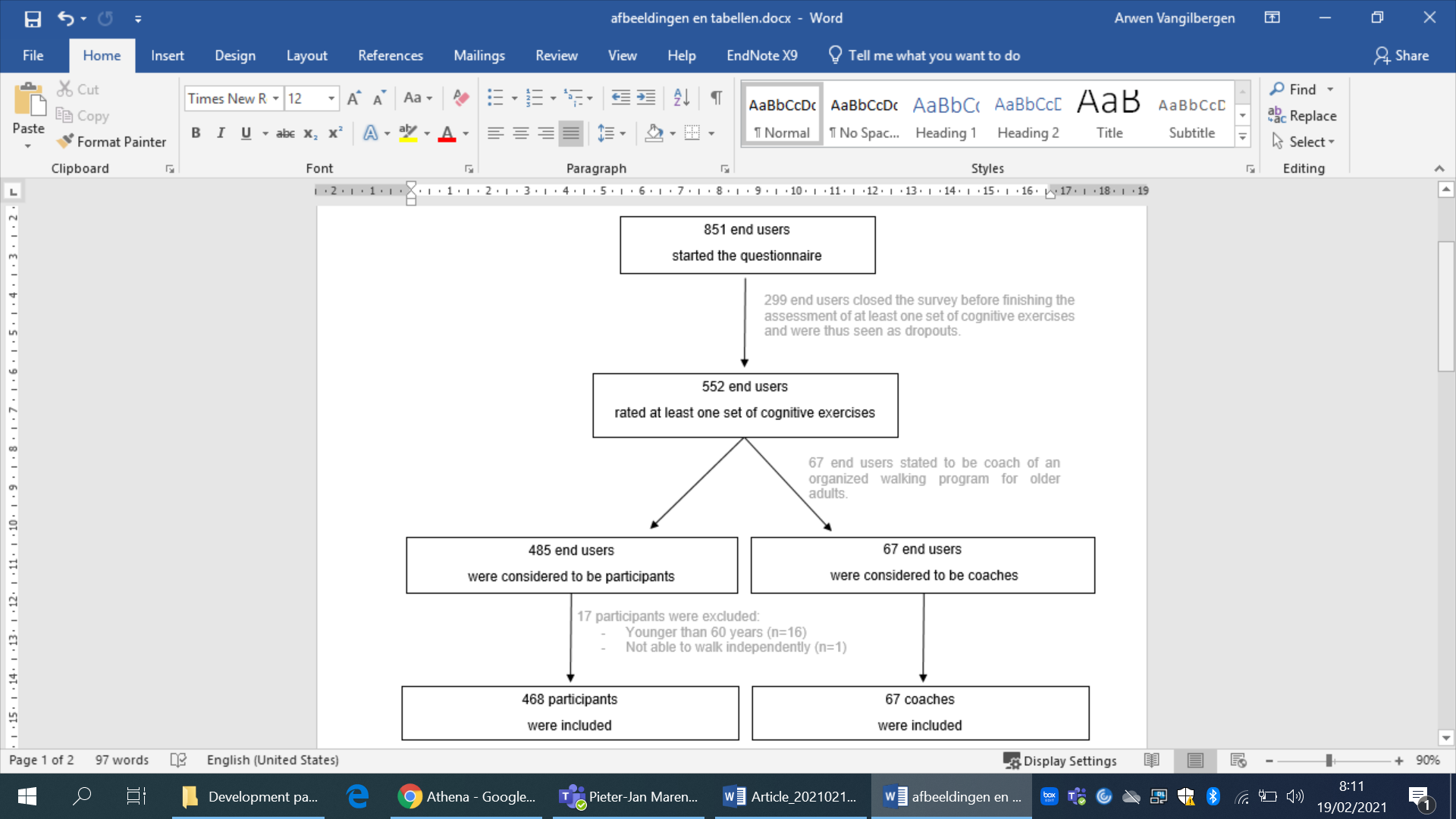

Supplement: Supplementary file 9 — Additional file 9. Flow chart of end users in the survey. [file 12877_2022_2823_MOESM9_ESM.docx]
